# Current Distribution and Diagnostic Features of Two Potentially Invasive Asian Buprestid Species: *Agrilus mali* Matsumura and *A. fleischeri* Obenberger (Coleoptera: Buprestidae)

**DOI:** 10.3390/insects11080493

**Published:** 2020-08-02

**Authors:** Mark G. Volkovitsh, Alexey V. Kovalev, Marina J. Orlova-Bienkowskaja

**Affiliations:** 1 Zoological Institute, Russian Academy of Sciences, Moscow 119991, Russia; 2 All-Russian Research Institute of Plant Protection, St. Petersburg 168084, Russia; melasis313@gmail.com; 3 A.N. Severtsov Institute of Ecology and Evolution, Russian Academy of Sciences, Moscow 119991, Russia; marinaorlben@yandex.ru

**Keywords:** jewel beetles, alien species, biological invasions, insects, pests, apple trees, poplars, Asia, native ranges

## Abstract

**Simple Summary:**

Knowledge of the diagnostic features and native ranges of invasive pests is vital for their correct identification and monitoring. In this regard, the diagnostic characters and geographical ranges of two potentially invasive Asian buprestid species: the quarantine apple tree pest, *Agrilus mali* Matsumura, and the poplar pest *A. fleischeri* Obenberger are studied and analyzed. Based on the examination of museum collections and literature sources, the diagnostic characters to distinguish both species from their congeners are discussed, the comprehensive databases of records of the exact collecting sites are compiled, and detailed maps of their ranges are generated. Occurrence of *A. mali* in Japan is not confirmed. Outbreak sites of *A. mali* in Xinjiang most likely represent the newly forming invasion areas; their proximity to the wild apple stands in the Kazakh part of the Tien Shan is a direct threat to Kazakhstan and adjacent countries. Sites damaged by *A. fleischeri* in Liaoning are situated within its native range; the outbreaks were likely triggered by the switch from indigenous to introduced poplars. The results of the study will facilitate the correct identification and monitoring of the pests in case of their findings in new areas.

**Abstract:**

Our goal is to analyze the known geographical ranges and diagnostic features of two potentially invasive Asian buprestid species: the quarantine apple tree pest, *Agrilus mali* Matsumura, and the poplar pest *A. fleischeri* Obenberger. Based on the examination of museum collections and literature sources, we compiled comprehensive databases of records of the exact collecting sites for both species and generated detailed maps of their ranges. There are 51 documented localities for *A. mali* in the Russian Far East and East Siberia, Mongolia, China, and the Korean peninsula, and there are 53 documented localities for *A. fleischeri* in the Far East and Siberia, Kazakhstan, Mongolia, China, and Japan. No evidence of the presence of *A. mali* in Japan was found. Outbreak sites of *A. mali* in Xinjiang in the 2000s most likely represent the newly forming invasion areas; their proximity to the wild apple stands in the Kazakh part of the Tien Shan is a direct threat to Kazakhstan and adjacent countries. Sites damaged by *A. fleischeri* in Liaoning are situated within its native range; the outbreaks were likely triggered by the switch from indigenous to introduced poplars. This situation is similar to the early stages of emerald ash borer (*Agrilus planipennis* Fairmaire) invasion.

## 1. Introduction

Knowledge of the native range of invasive pests is vital for their monitoring, understanding their biology, modeling potential invasive distribution, and searching for natural enemies for biological control programs [1,2,3]. Followed by the devastating and costly invasion of the emerald ash borer, *Agrilus planipennis* Fairmaire, 1888, into North America [4,5,6] and European Russia [7,8,9], there was an increased interest in other representatives of extremely speciose (more than 3000 species) genus *Agrilus* Curtis, 1825 (Coleoptera: Buprestidae: Agrilinae) [10], which pose the real or potential threat of massive outbreaks and long-distance invasions. In particular, the North American bronze birch borer *Agrilus anxius* Gory, 1841, is a quarantine pest included on the A1 quarantine list of the European and Mediterranean Plant Protection Organization (EPPO, Paris, France) [11]. Among representatives of the genus *Agrilus*, two Asian species with high outbreak and range expansion potential also attract particular attention: the quarantine pest of apple and some other fruit trees, an apple buprestid *Agrilus mali* Matsumura, 1924, and the poplar pest *A. fleischeri* Obenberger, 1925 [12,13,14]. *Agrilus mali* is on the quarantine lists of the Russian Federation, Eurasian Economic Union [15], and EPPO [16]. *Agrilus fleischeri* is on the A2 list of pests recommended for regulation as quarantine pests [17].

*Agrilus mali* was originally described as a destructive pest of cultivated apple trees in Korea [18], and the author suggested that it was introduced to Korea from East China (Liaoning) via seedlings of apple trees. Since then, it has been regarded as a dangerous quarantine pest in the Russian Far East, East Siberia, and East China ([19], with bibliography). According to Chinese authors, in 1993, *A. mali* was introduced in the Ili River valley (Xinjiang Uyghur autonomous region, Xinjiang) from Shandong via apple seedlings and later caused extensive tree mortality in the wild apple (*Malus sieversii* (Ledeb.) Roem.) forests in the Tien Shan Mountains [12,13,20,21,22]. *Malus sieversii* is a wild apple tree species native to Central Asia and distributed in Kazakhstan, Kyrgyzstan, Tajikistan, Uzbekistan, Afghanistan, and Northwestern China. It is regarded as a key ancestor of the domestic apple tree [12,13,22]. Subsequently, *A. mali* has killed millions of wild apple trees and infested more than 80% of the total area of wild apple forests in the Ili valley [20,21,22]. References to the presence of *A. mali* in Japan [12,13,23,24] are questionable because no records were found in the catalogues and faunal lists of Japanese Buprestidae.

*Agrilus fleischeri* is also native to Asia but its known range is much more extensive than that of *A. mali* and includes West Siberia and East Kazakhstan [19,25]. In Russian literature, *A. fleischeri* is frequently cited as a subspecies of the well-known European poplar pest *A. ater* (Linnaeus, 1767) [24]. Recently, *A. fleischeri* caused severe damage in poplar tree plantations, especially the nonindigenous cultivar *P. nigra* var. *italica* in the Liaoning province of China [14].

Thus, *A. mali* and *A. fleischeri* can be regarded as potential invaders posing a real threat to native and cultivated woody host plants not only within but also outside their native ranges. To develop preventive measures against their further distribution, to model possible mechanisms of invasion, and to identify effective measures of control, including biological control agents, it is necessary first to study the native ranges of potential invaders. The knowledge of diagnostic features is also crucial for the timely identification of the potential invaders.

## 2. Materials and Methods

To study a distribution of two *Agrilus* species, we compiled databases comprising 51 localities of *A. mali* and 53 localities of *A. fleischeri* in East Eurasia, totaling 104 documented localities (see Table 1 and Table 2, and Supplementary Table S1), and made detailed maps of their current ranges using DIVA-GIS 7.5 software [26]. Images were obtained using a Canon EOS 40D digital camera with a Canon MP-E-65 mm objective (Tokyo, Japan) and montaged using Helicon Focus software (Helicon Soft Ltd., Kharkiv, Ukraine) (A.V. Kovalev).

The sources of information were mainly the specimens deposited in the collections of the Zoological Institute of Russian Academy of Sciences (ZIN, St. Petersburg, Russia) and the National Museum (NMPC, Prague, Czech Republic) (type specimens of *A. jenissejensis* Obenberger, 1924, *A. fleischeri* Obenberger, 1925, and *A. fleischeri kurosawai* Obenberger, 1940). Additional information on the documented localities of *A. mali* and *A. fleischeri* was compiled from the personal communications of collection curators [27,28,29,30,31,32,33] and literature sources [12,13,14,18,19,25,34,35,36,37,38,39,40,41,42,43,44].

To confirm or exclude the presence of *A. mali* in Japan, as reported in some publications [12,13,23,24], we also examined the available material and literature data on the closely related species from the subgenus *Sinuatiagrilus* Alexeev, 1998 (*sinuatus* species-group), from the Russian Far East, China, and Japan (*A. mendax* Mannerheim, 1837; *A. sachalinensis* Obenberger, 1935; *A. sinuatus yokoyamai* Iga, 1955; *A. zhelochovtsevi* Alexeev, 1979), and we also consulted with Japanese buprestid expert Tahakaru Hattori [32].

Terminology used in the morphological descriptions follows that reported in [19,45].

## 3. Results

### 3.1. Taxonomy, Distribution, and Host Plants of Agrilus mali and A. fleischeri

#### 3.1.1. *Agrilus mali* Matsumura, 1924, Apple Buprestid

**Figure 1:**
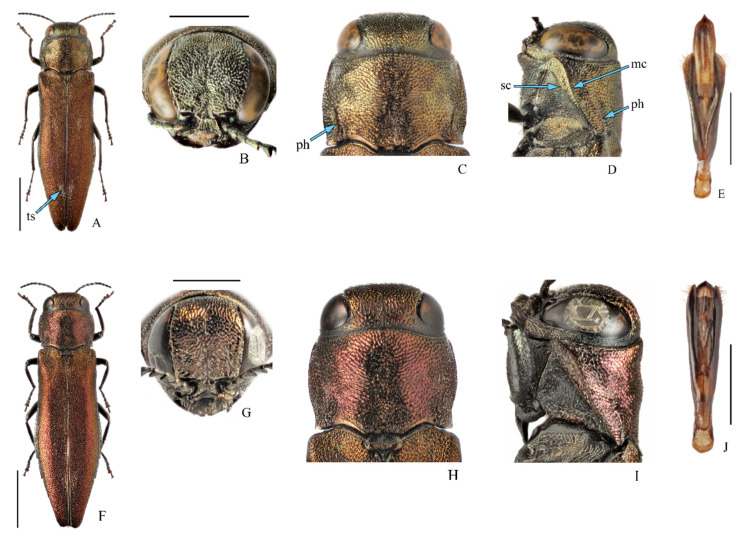
(**A**–**E**)—*Agrilus mali* Matsumura, 1924, male. (**A**)—Habitus; (**B**)—head; (**C**)—pronotum, dorsal view; (**D**)—same, lateral view; (**E**)—aedeagus, dorsal view. (**F**–**J**)—*Agrilus sinuatus sinuatus* (Olivier, 1790), male. (**F**)—Habitus; (**G**)—head; (**H**)—pronotum, dorsal view; (**I**)—same, lateral view; (**J**)—aedeagus, dorsal view. **mc**—marginal carina, **ph**—prehumerus, **sc**—submarginal carina, **ts**—tomentose spots. (**A**–**E,J**)—Original, (**F**–**I**)—after [45]. Scale bars for (**A**,**F**)= 2.0 mm; for (**B**–**D**),(**G**–**I**) = 1.0 mm; for (**E**,**J**) = 0.5 mm. Photo A.V. Kovalev.

**Figure 2:**
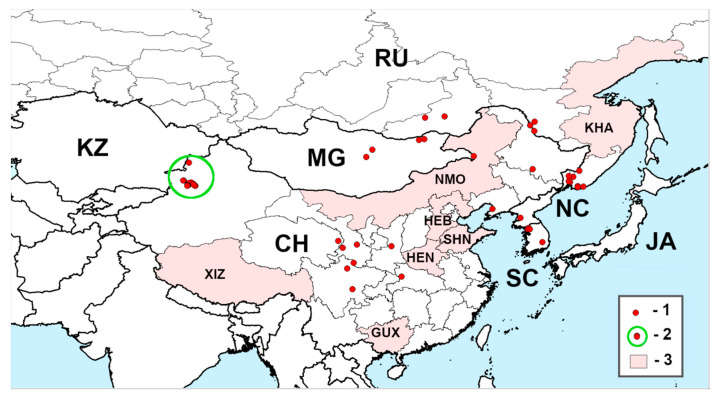
Range of *Agrilus mali* Matsumura, 1924. 1—documented localities, 2—outbreak sites of *Malus sieversii* in Xinjiang Uyghur autonomous region, 3—reported administrative units for which exact localities were not found. CH—China, JA—Japan, KZ—Kazakhstan, MG—Mongolia, NC—North Korea, RU—Russia, SC—South Korea; GUX—Guangxi, HEB—Hebei, HEN—Henan, KHA—Khabarovskii Krai, NMO—Nei Mongol, SHN—Shandong, XIZ—Xizang.

Figure 1A–E and Figure 2; Table 1.

Synonym: *jenissejensis* Obenberger, 1924.

Taxonomic position: subgenus *Sinuatiagrilus* Alexeev, 1998; *sinuatus* species-group Jendek, Grebennikov, 2011.

Diagnosis. Body (Figure 1A): length 6.1–8.7 mm; unicolor, metallic, coppery-red, underside also copper; elytra with three pairs of white tomentose spots. Head (Figure 1B,C): frons and vertex with longitudinal medial impression, vertex slightly depressed or flat as seen from above, wide, slightly wider than frons above antennal fossae, frons with almost regularly arcuate sides, very densely punctate. Pronotum (Figure 1C,D): widest at mid-length, sides arcuately converging toward anterior angles and base, slightly emarginated basally, posterior angles nearly rectangular; disc without distinct medial impressions; marginal and submarginal carinae (Figure 1D: mc, sc) almost touching each other or even merging posteriorly; prehumeri (Figure 1C,D: ph) well-marked, strongly elevated, arcuate from above but subparallel to common part of marginal carinae in lateral view, extending to approximately mid-length of pronotum. Elytra (Figure 1A): coppery-red, matt, with three pairs of white tomentose spots (Figure 1A: ts) of setiform scales located in humeral depressions and along the suture in anterior and posterior thirds, two anterior pairs frequently missing or poorly marked, posterior sutural pair always well-marked, cuneiform; apices arcuate or slightly angulate, with very finely denticulate margins. Pygidium: rounded, without apical process. Abdominal ventrites without tomentose spots. Aedeagus (Figure 1E): tegmen widest in apical half, parameres apically with wide membranous marginal area; penis nearly subparallel with apex strongly acute. Larva: habitus is illustrated in Matsumura [18], but it is impossible to find any characteristics to distinguish it from other congeners.

Comparison. The most important diagnostic characteristics of *A. mali* to distinguish it from other members of subgenus *Sinuatiagrilus* (*sinuatus* species-group) are the presence of three pairs of tomentose spots on elytra and an aedeagus structure. Additionally, it differs from *A. sinuatus* (Olivier) (Figure 1F–J), which bears a single pair of poorly marked tomentose spots in the posterior third of elytra, better developed and numerous elytral spots and an aedeagus structure (in *A. sinuatus* apical membranous areas on parameres nearly lacking and penis blunted apically). Compared with *A. sachalinensis* Obenberger, it differs based on its smaller size and the presence of white tomentose spots on elytra. Compared with *A. zhelochovtsevi* Alexeev, it differs based on the presence of tomentose spots on elytra and an absolutely different aedeagus structure.

Note. According to Jendek [19,46], *A. zhelochovtsevi* is a synonym of *A. sinuatus sachalinensis*. However, in our opinion, supported by comparison of male genital structures and external morphological characteristics, *A. zhelochovtsevi*, *A. sinuatus*, and *A. sachalinensis* belong to three different species, and subgenus *Sinuatiagrilus* needs a taxonomic revision.

Host plants: *Malus* spp.; *M. domestica* Borkh., *M. pumila* Mill., *Pyrus* sp. (Rosaceae) [47]; *Malus spectabilis* (Aiton) Borkh., *M. prunifolia* (Willd.) Borkh., *M. asiatica* var. *rinki* (Koidz.) H. Ohle, *M. baccata* (L.) Borkh, *Sorbus* sp., *Prunus* sp. (cherry), *Cydonia oblonga* Mill. (Rosaceae), *Emmenopterys henryi* Oliv. (Rubiaceae) [48]; *Malus sieversii* (Ledeb.) M. Roem. (Xinjiang, introduction) [12,13,49]. References to *Juglans* and *Salix* are erroneous [19]; references for *Emmenopterys* require verification. Data for *Sorbus* can refer to *A. mendax* Mannerheim, 1837.

General distribution. Russia (East Siberia, Far East), Mongolia, China (Gansu, Guangxi, Hebei, Heilongjiang, Henan, Hubei, Nei Mongol, Qinghai, Sichuan, Shandong, Xinjiang, Xizang), North Korea, and South Korea [46].

Documented distribution. As can be seen from the map compiled from 51 documented localities and records from literature sources (Figure 2; Table 1), *A. mali* is widely distributed throughout continental East Asia, from the Russian Far East (Primorskii Krai, Amurskaya Oblast) and the Korean Peninsula (Pyongyang in North Korea, Gyeonggi-do, Gyeongsangbuk-do in South Korea) on the east to East Siberia (Transbaikalia, Chitinskaya Oblast), which is the northern part of the range; Central Mongolia (Dornod, Tuv) and Gansu Province of China on the west, and Sichuan province on the south. However, there are no documented localities or records from Japan. We could not find the exact localities of *A. mali* within some administrative units of Russia (Khabarovskii Krai) and China (Guangxi, Hebei, Henan, Nei Mongol, Shandong, Xizang) although *A. mali* was reported from these territories [19,46].

#### 3.1.2. *Agrilus fleischeri* Obenberger, 1925

**Figure 3:**
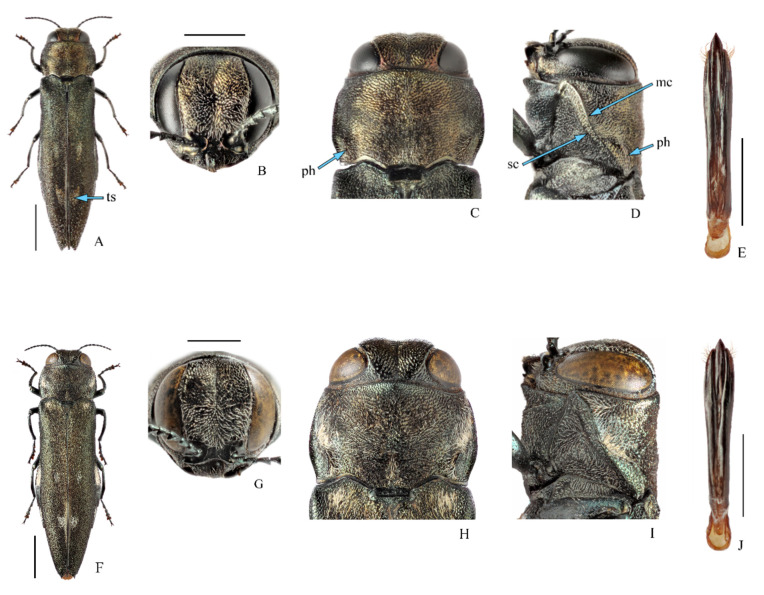
(**A**–**E**)—*Agrilus fleischeri* Obenberger, 1925, (**A**–**D**)—female, (**E**)—male. (**A**)—Habitus; (**B**)—head; (**C**)—pronotum, dorsal view; (**D**)—same, lateral view; (**E**)—aedeagus, dorsal view. (**F**–**J**)—*Agrilus ater* (Linnaeus, 1767), male. (**F**)—Habitus; (**G**)—head; (**H**)—pronotum, dorsal view; (**I**)—same, lateral view; (**J**)—aedeagus, dorsal view. **mc**—marginal carina, **ph**—prehumerus, **sc**—submarginal carina, **ts**—tomentose spots. (**A**–**E,J**)—original, (**F**–**I**)—after [45]. Scale bars for (**A**,**F**) = 2.0 mm; for (**B**–**D**,**G**–**I**) = 1.0 mm; for (**E**,**J**) = 0.5 mm. Photo A.V. Kovalev.

**Figure 4:**
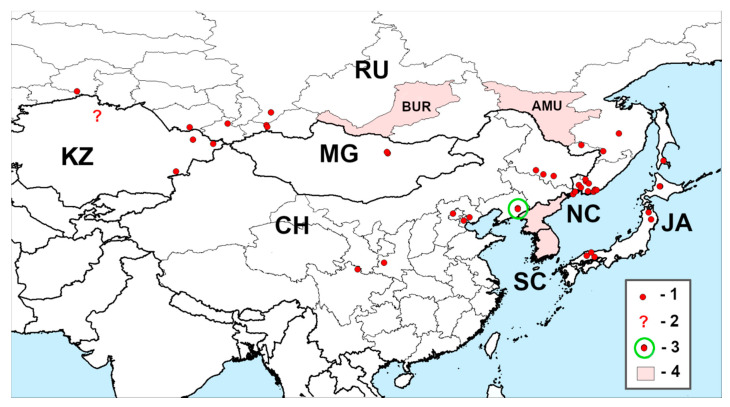
Range of *Agrilus fleischeri* Obenberger, 1925. 1—documented localities, 2—specimen with doubtful identification from North Kazakhstan, 3—outbreak site in Liaoning, 4—reported countries and administrative units for which exact localities were not found. CH—China, JA—Japan, KZ—Kazakhstan, MG—Mongolia, NC—North Korea, RU—Russia, SC—South Korea, AMU—Amurskaya Oblast, BUR—Buryatia Republic.

Figure 3A–E and Figure 4; Table 2.

Synonyms: *kochi* Théry, 1942; *fleischeri kurosawai* Obenberger, 1940; *fleischeri nipponicola* Kurosawa, 1963; *tscherepanovi* Stepanov, 1954.

Taxonomic position: subgenus *Uragrilus* Semenov, 1935; *spinipennis* species-group Jendek and Grebennikov, 2011.

Diagnosis. Body (Figure 3A): length 7.3–12.0 mm; slightly bicolored, head and pronotum black with metallic, greenish-blue (male), or bronze (female) tint; elytra blackish, occasionally with greenish (male) or bronze (female) tint, bearing three pairs of white tomentose spots. Head (Figure 3B,C): frons and vertex with well-marked longitudinal medial impression, vertex deeply depressed as seen from above, narrow, slightly wider (male), or narrower (female) than frons above antennal fossae, frons widest in anterior third, densely covered with concentric punctate grooves. Pronotum (Figure 3C,D): widest at mid-length, sides converging toward anterior angles and base, posterior angles obtuse, sides with incurved tomentose spots; disc with shallow medial impressions; marginal and submarginal carinae (Figure 3D: mc, sc) subparallel, distinctly separated; prehumeri (Figure 3C,D: ph) well-marked, strongly elevated, arcuate, extending to approximately mid-length of pronotum. Elytra (Figure 3A): blackish with greenish or bronze tint, matt, bearing three pairs of white tomentose spots (Figure 3A: ts) of setiform scales located in humeral depressions, in approximately mid-length and closely to suture in posterior third, posterior spots cuneiform, in shape of “V”; surface between anterior and middle spots and preapical third with rather dense white setiform scales; apices acutely angulate, with serrate margins. Pygidium with apical process. Abdominal ventrites and laterosternites with tomentose spots. Aedeagus (Figure 3E): parameres subparallel, apical membranous area poorly marked; penis with acute apex, narrow, and subparallel. Larva: not described.

Comparison. In habitus, *A. fleischeri* is very similar to European poplar pest *A. ater* (Linnaeus, 1767) (Figure 3F–J). It was regarded as its subspecies in the Russian literature [24], but most studies treated *A. fleischeri* as a distinct species [19,46]. *Agrilus ater* differs from *A. fleischeri* based on a nearly unicolor blackish-bronze or blackish body with more contrast tomentose spots, marginal and submarginal carinae distinctly converging and nearly touching each other in posterior half, posterior pair of elytral spots arranged transversally not forming “V”, elytral apices extended into acute denticle, aedeagus slightly expanded in apical half. Compared with other *Agrilus* species, it differs based on a combination of rather large body size (up to 12 mm), blackish coloration with white tomentose spots, presence of pygidial process, male genital structures, and some other characteristics [19,24].

Host plants: *Populus laurifolia* Ledeb., *P. sieboldii* Miq., *Salix schwerinii* E.L. Wolf (Salicaceae) (synopsis) [47]; *Populus davidiana* Dode, *P. nigra* var. *italica* Munchh. (introduced) [14].

General distribution (Figure 4; Table 2): Russia (West Siberia, East Siberia, Far East), Kazakhstan, Mongolia, China (Beijing, Heilongjiang, Liaoning, Sichuan, Shaanxi), North Korea, South Korea, and Japan [19,46].

Documented distribution. The range of *A. fleischeri* is much more extensive than that of *A. mali* (Figure 2). It extends from Japan (Hokkaido, Honshu) and the Russian Far East (Sakhalin, Primorskii and Khabarovskii Krais, Evreiskaya Avtonomnaya Oblast) on the east and throughout Northeast and Central China (Heilongjiang, Liaoning, Beijing, Sichuan, Shaanxi), Mongolia (Tuv), Tyva Republic and the Altai Mountains in Russia, and East Kazakhstan to North Kazakhstan and West Siberia (Tyumenskaya Oblast). We could not find the exact localities of *A. fleischeri* within Amurskaya Oblast and Buryatia Republic (Russia), North Korea, and South Korea although it was reported in these territories [19,46].

Note. In the westernmost part, the range of *A. fleischeri* comes close but does not overlap with the range of European *A. ater*. The latter is distributed throughout almost all of Europe but its eastern limits are still poorly known. According to the materials in ZIN, the easternmost locations of this species are situated in Northwest Kazakhstan (Oral Oblast, former Uralsk), Orenburgskaya Oblast, and near Irgizla in Bashkortostan, Russia. It can be assumed that the eastern boundaries of the *A. ater* range are the Ural Range and the Ural River. The identification of the specimen from North Kazakhstan [38] needs verification; however, in our view, it is rather *A. fleischeri* than *A. ater* based on the findings of the first in closely situated Tyumenskaya Oblast of Russia [25].

## 4. Discussion

The numerous documented localities of *A. mali* in the Xinjiang Uyghur autonomous region of China, in which massive outbreaks in the wild apple, *Malus sieversii*, were observed [12,13,20,22], are situated rather far from its main range and can be regarded as a newly forming invasion area which indicates the expansion of its range. In addition, the type localities of *A. mali* in the Korean Peninsula fit its native range well, which contradicts the hypothesis that it was introduced there together with the apple seedlings from Liaoning province of China [18].

We failed to find any evidence confirming the occurrence of *A. mali* in Japan as some sources report [12,13,23,24] and we treat such reports as false indications.

The proximity of the invasion hotspot of *A. mali* in the Ili River valley in the Xinjiang Uyghur autonomous region of China to the indigenous stands of *M. sieversii* in the Kazakh part of the Tien Shan means a direct threat to the forest resources of the adjacent Kazakhstan and the other countries of Central Asia.

Outbreak sites of *A. fleischeri* in the Liaoning province of China [14] are situated within the eastern part of its range. In this case, the local switch of the pest from indigenous (*Populus davidiana*) to introduced (*P. nigra* var. *italica*) poplars with a subsequent outbreak is more likely than invasion or range expansion. The current situation with *A. fleischeri* is rather similar to the early stages of the invasion of the emerald ash borer, *Agrilus planipennis*. First, it switched from the indigenous host on the cultivated nonresistant American ashes in Eastern China and the Russian Far East [1,51], serving as a primary reason that triggered the local outbreaks. Then, it was imported together with wood packing material to North America and European Russia, and it seems that its further distribution is limited by only minimal winter temperatures [52] and the range of ash trees [53,54].

## 5. Conclusions

1. Distributional maps for two potentially invasive buprestid species, *Agrilus mali* and *A. fleischeri*, are compiled based on the documented records for 51 and 53 localities, correspondingly.
2. A dangerous quarantine pest of wild and cultivated apple trees, *A. mali*, is distributed over the continental East Asia from the Russian Far East, Northeast China, and the Korean Peninsula to Mongolia and Eastern Siberia; however, its occurrence in Japan is not confirmed.
3. Massive outbreak sites of *A. mali* on *Malus sieversii* in Xinjiang likely represent a newly forming invasion area, which indicates the expansion of its range; their proximity to the wild apple stands in the Kazakh part of the Tien Shan is a direct threat to Kazakhstan and other Central Asian countries.
4. *Agrilus fleischeri* is more widely distributed in the Russian Far East and Siberia, Kazakhstan, Mongolia, China, and Japan than was assumed earlier; its westernmost limits come close but do not overlap with the range of the closely related European poplar pest *A. ater*.
5. Outbreak sites of *A. fleischeri* in Liaoning (China) are situated within its range; the local switch from indigenous to introduced poplars is the most likely reason for the outbreak. Thus, there is no reason to claim an expansion of its native range. The situation is similar to the early stages of emerald ash borer invasion and *A. fleischeri* should be monitored as a potential invasion threat.

## Figures and Tables

**Table 1:** Documented records of *Agrilus mali* Matsumura, 1924. CH—China, MG—Mongolia, NC—North Korea, RU—Russia, SC—South Korea, ZIN—specimens in ZIN.

Region	Number of Mapped Localities	Years of Collection	Host Plants	Sources of Information
RU: Primorskii Krai	10	1926–1977	*Malus* sp. (part.)	ZIN; [28,44]
RU: Khabarovskii Krai	0	-	-	[19]
RU: Amurskaya Oblast	3	1927, 1958	-	ZIN; [19]
RU: Zabaikalskii Krai	1	1898	-	ZIN
RU: Chitinskaya Oblast	2	1923, 1924	*Malus* sp.	ZIN; [27]
MG: Dornod	3	1976	Wild *Malus* sp.	ZIN
MG: Tuv	2	1976	Wild *Malus* sp.	ZIN
CH: Heilongjiang	1	1931	-	ZIN
CH: Liaoning	1	-	-	[18]
CH: Xinjiang	15	2011–2017	*M. sieversii*, *M. domestica*	[12,13,35,37]
CH: Gansu	2	1992, 1996	-	[19]
CH: Qinghai	1	2008	-	[35]
CH: Hubei	1	2002	-	[19]
CH: Sichuan	3	1990–2001	-	[19]
CH: Shaanxi	1	2006	-	[35]
CH: Guangxi	0	-	-	[19]
CH: Hebei	0	-	-	[19]
CH: Henan	0	-	-	[19]
CH: Nei Mongol	0	-	-	[19]
CH: Shandong	0	-	-	[19]
CH: Xizang	0	-	-	[19]
NC: Pyongyang	1	-	*M. domestica*	[18]
SK: Gyeonggi-do	1	-	*M. domestica*	[18]
SK: Taegu	1	-	*M. domestica*	[18]
SK: Inchon	1	-	*M. domestica*	[18]
SK: Seoul	1	1940	-	[32]
	51	1898–2017		

**Table 2:** Documented records of *Agrilus fleischeri* Obenberger, 1925. CH—China, JA—Japan, KZ—Kazakhstan, MG—Mongolia, NC—North Korea, RU—Russia, SC—South Korea, ZIN—specimens in ZIN; ^1^—determination needs verification.

Region	Number of Mapped Localities	Years of Collection	Host Plants	Sources of Information
RU: Sakhalin	1	1930	-	[19]
RU: Primorskii Krai	18	1899–2015	-	ZIN; [19,27,28,30]
RU: Khabarovskii Krai	2	1976	-	ZIN
RU: Evreiskaya AO	1	-	-	ZIN
RU: Amurskaya Oblast	0	-	-	[19]
RU: Byrjatia Republic	0	-	-	[19]
RU: Tuva Republic	2	1949, 1979	*Populus* sp.	[19,27]
RU: Altai Republic	1	1909	-	ZIN
RU: Altaiskii Krai	1	1910	-	ZIN
RU: Tyumenskaya Oblast	1	2002	-	[25]
RU: Krasnoyarskii Krai	1	1989	-	[27]
KZ: Almaty Oblast	1	2016	*Populus tremula*	ZIN; [42]
KZ: Vostochno-Kazakhstanskaya Oblast	2	2001, 2009	*Populus* sp.	[19,43]
KZ: Akmolinskaya Oblast ^1^	1	2002	-	[38]
MG: Tuv	2	2003	-	[19]
CH: Heilongjiang	5	1931, 1939, 1940	-	ZIN; [19]
CH: Liaoning	2	2013–2015	*Populus davidiana, P. nigra* var. *italica*	[14,50]
CH: Beijiing	2	2000 (1)	*Populus* spp. in plantations; *Salix* sp.	[14,19]
CH: Hebei	1	-	*Populus* spp. in plantations; *Salix* sp.	[14]
CH: Shaanxi	1	1998	-	[19]
CH: Sichuan	1	2001	-	[19]
NC	0	-	-	[19]
SC	0	-	-	[19]
JA: Honsu	5	1996 (1)	-	[19,34,39,40]
JA: Hokkaido	2	2015 (1)	-	[19,36]
	53	1899–2016		

## Supplementary Materials

The following are available online at https://www.mdpi.com/2075-4450/11/8/493/s1, Table S1: Current distribution and diagnostic features of two potentially invasive Asian buprestid species: Agrilus mali Matsumura and A. fleischeri Obenberger (Coleoptera: Buprestidae).
